# Epigenetic Modulating Chemicals Significantly Affect the Virulence and Genetic Characteristics of the Bacterial Plant Pathogen *Xanthomonas campestris* pv. *campestris*

**DOI:** 10.3390/genes12060804

**Published:** 2021-05-25

**Authors:** Miroslav Baránek, Viera Kováčová, Filip Gazdík, Milan Špetík, Aleš Eichmeier, Joanna Puławska, Kateřina Baránková

**Affiliations:** 1 Mendeleum—Institute of Genetics, Faculty of Horticulture, Mendel University in Brno, 69144 Lednice, Czech Republic; filip.gazdik@zf.mendelu.cz (F.G.); milan.spetik@mendelu.cz (M.Š.); ales.eichmeier@mendelu.cz (A.E.); katerina.barankova@mendelu.cz (K.B.); 2 Institute for Biological Physics, University of Cologne, 50923 Köln, Germany; vkovacov@uni-koeln.de; 3 Department of Phytopathology, Research Institute of Horticulture, 96-100 Skierniewice, Poland; joanna.pulawska@inhort.pl

**Keywords:** bacterial epigenetics, DNA methylation, virulence, *Xanthomonas campestris*, dual RNA-seq

## Abstract

Epigenetics is the study of heritable alterations in phenotypes that are not caused by changes in DNA sequence. In the present study, we characterized the genetic and phenotypic alterations of the bacterial plant pathogen *Xanthomonas* *campestris* pv. *campestris* (*Xcc*) under different treatments with several epigenetic modulating chemicals. The use of DNA demethylating chemicals unambiguously caused a durable decrease in *Xcc* bacterial virulence, even after its reisolation from infected plants. The first-time use of chemicals to modify the activity of sirtuins also showed some noticeable results in terms of increasing bacterial virulence, but this effect was not typically stable. Changes in treated strains were also confirmed by using methylation sensitive amplification (MSAP), but with respect to registered SNPs induction, it was necessary to consider their contribution to the observed polymorphism. The molecular basis of the altered virulence was deciphered by using dualRNA-seq analysis of treated *Xcc* strains infecting *Brassica* *rapa* plants. The results of the present study should promote more intensive research in the generally understudied field of bacterial epigenetics, where artificially induced modification by epigenetic modulating chemicals can significantly increase the diversity of bacterial properties and potentially contribute to the further development of the fields, such as bacterial ecology and adaptation.

## 1. Introduction

In general, epigenetics is the study of alterations in the features of organisms that are not caused by variations in their DNA sequence. These kinds typically result from stable shifts in gene expression caused by modifications of the nucleotides in DNA, e.g., methylation, non-coding RNA interference, or alterations in eukaryotic chromatin [1]. The study of epigenetics has attained the greatest success in the field of human medicine, where it helps to cure some kinds of cancer or predispositions to certain behaviours [2,3,4]. Many important discoveries have been made in plants in terms of their adaptation to stress and transfer of gained features to the next generation [5,6]. However, little is known regarding the role of epigenetics in bacterial physiology, with only a few studies having investigated this topic.

Compared to eukaryotes, prokaryotic organisms clearly have a more limited spectrum of epigenetic modifications due to the lack of chromatin. However, bacteria still have several factors that contribute to the overall epigenetic state of the cell. The most famous of these factors are DNA-methylation based epigenetic markers, which are able to modify gene expression in both eukaryotic and prokaryotic organisms. C^5^-Methyl-cytosine (m5C) is the most used modification of the standard bases in eukaryotic DNA, and this modification is also observed in prokaryotic DNA. Additionally, bacterial genomes contain N^6^-methyl-adenine (m6A), which is also present in lower eukaryotes but not in vertebrates, and N^4^-methyl-cytosine (m4C), which is exclusive to bacteria [7]. The synthesis of m6A, m4C, and m5C is catalyzed by DNA methyltransferases (DNMTs), with S-adenosyl-methionine serving as a methyl group donor [8]. DNMTs are divided into two groups. The first group is represented by transferases active in restriction-modification (R-M) systems, where the methylation of the specific base inhibits the activity of restriction endonucleases activity and protects the host cell against foreign DNA, e.g., phages [9]. The second group consists of solitary (“orphan”) DNA methyltransferases not associated with R-M systems, which are assigned by housekeeping functions [10]. Recently, the traditional view of this distinction has begun to change, as more roles for DNMT included in R-M systems are described [11] or solitary DNA methyltransferases show higher abundancy than expected [12].

In terms of epigenetics, it is advantageous that a variety of chemicals capable of modifying epigenetic information have been described. One of the best-known examples is azacytidine, an analogue of cytidine that specifically inhibits DNA methylation by sequestering DNMTs. Azacytidine has been successfully used in human medicine [13,14] and for the confirmation of the role of DNA methylation in the plant adaptation process [15]. In addition to changes in DNA methylation, changes in the frequency of point mutations can theoretically be expected after Azacytidine treatment, given the fact that DNA methylation plays a role in the bacterial mismatch repair system [16]. In bacteria, such a directly focused study is lacking, but the effect of Azacytidine treatment on the increased number of point mutations was confirmed by Kiselev et al. [17] in plants. One of the most commercially successful analogues of azacytidine is zebularine, which shows higher stability and does not inhibit RNA synthesis [18].

Other chemicals that can potentially influence epigenetic information are sirtuins, a family of intracellular enzymes capable of catalyzing the β-nicotinamide adenine dinucleotide (β-NAD^+^)-dependent N^ε^-acyl-lysine deacylation of histones and non-histone protein substrates [19]. As deacetylating agents have been shown to regulate many biological processes in cell metabolism, including DNA repair, inflammatory responses, the cell cycle, and apoptosis [20], the functional properties of sirtuins were summarized in a review by Kosciuk et al., with an emphasis on epigenetics [21]. Until the 1990s, acetylation was primarily studied in eukaryotic organisms in the context of chromatin maintenance and gene expression. Currently, it is even more evident that bacterial protein acetylation, including within the nucleoid (a bacterial cell region containing a vast majority of genetic material, RNA, and proteins) also plays a prominent role in bacterial primary and secondary metabolism, virulence, transcription, and translation [22]. Most bacterial sirtuins described to date belong to the CobB type, a bacterial homologue of yeast Sir2. Sir2 homologs can also be detected in all of the sequenced genomes of *Xanthomonas campestris* pv. *campestris* (Pammel) Dowson currently available at NCBI, with these genes being under Acc. Nos. CAP49662.1 or AAM39625.1.

The *Xcc* was selected as a model organism to study the effects of epigenetic modulating chemicals on bacteria. This bacterium causes black rot disease of crucifers, which is a worldwide problem of huge economic importance. *Xcc* is even rated as one of the top 10 economically most serious plant pathogens in the world [23], but despite its economic importance, information on its genome methylation patterns is not yet available. To date, only one paper has been published on genome methylation in *Xanthomonas* pathogens, a study in which the methylation patterns of cytosines and adenines in the genomes of *X. axonopodis* pv. *glycines* and *X. campestris* pv. *vesicatoria* were compared [24]. Regarding its microbiological characteristics, *Xcc* is an aerobic, gram-negative bacterium with a single polar flagellum. The current means of protection against this pathogen are highly limited, with the use of antibiotics in agriculture being generally undesirable. One crop protection strategy is to lower the virulence of bacterial strains.

The present study represents a genuine and complex investigation of the effects of a set of epigenetic modulating chemicals on a phytopathogenic bacterium, which was evaluated with respect to virulence, the DNA methylation-sensitive profiling of the treated strains, the gene expression of treated bacterial strains with different virulence phenotypes, and the associated plant transcriptome during host-pathogen interactions.

## 2. Materials and Methods

Ten epigenetic modulating chemicals (EMCs) were used for the treatment of the *Xcc* reference strain WHRI 1279A (Warwick HRI, Wellesbourne, UK), which was selected based on its previously proven course of infection on the *Brassica rapa* L. cultivar used in this study (Table 1). The mechanisms associated with the epigenetic effects of these compounds are different, but in general, they comprise five DNA methyltransferase inhibitors, four sirtuin inhibitors, and one sirtuin activator. Some of these compounds have well documented epigenetic effects, such as azacytidine (DNA demethylating agent), while some have recently described hypothetical effects. A list of used chemicals and a short description of their effects is presented in Table 1.

### 2.1. Xcc Treatment with Individual Epigenetic Modulating Chemicals

The *Xcc* reference strain WHRI 1279A was used as a reference strain throughout the experiment. A liquid culture of the *Xcc* strain was prepared in Mueller-Hinton broth (Sigma-Aldrich, Prague, Czech Republic) and diluted to a density of 10^5^ CFU·mL^−1^. Two milliliter aliquots of bacterial suspensions (BSs) in Mueller-Hinton broth were treated with individual EMC at concentration of 50 µM in broth, where 2.5% dimethyl sulfoxide (DMSO) was also present in the broth. Obtained cultures were further shaken (Jeio Tech shaker, Daejeon, Korea) for 24 h at 28 °C. Ten different epigenetically treated strains (ETSs) corresponding to ones listed in the Table 1 were prepared in this manner. Subsequently, 1 mL of culture for each ETS was transferred into 20 mL of EMC-free Mueller-Hinton broth to confirm the viability of the individual ETS. After another 36 h of culturing in the shaker (incubation on 28 °C) the ETSs were diluted to 10^8^ CFU·mL^−1^. Then, 1 mL of each ETS was used for DNA extraction used in subsequent downstream analysis through the Methylation Sensitive Amplified Polymorphism (MSAP) technique.

### 2.2. Plant Material, Inoculation with ETSs and Virulence Evaluation

Seedlings of *B. rapa* var. *pekinensis* ‘Hilton’ (MoravoSeed, Mušlov, Czech Republic) with two to four mature leaves were used for plant inoculation assays.

The frozen ETSs were recultivated in 20 mL of Mueller-Hinton broth, as mentioned above, and diluted to 10^8^ CFU·mL^−1^ (0.1 OD) in water. The plants were inoculated by spraying the leaves with 1 mL of ETS suspension per five plants (200 µL per plant). A set of 10 ETSs was used for the plant inoculation assay, together with a positive control (untreated *Xcc* strain WHRI 1279A), *Xcc* treated with 2.5% DMSO and a negative control (plants sprayed with water). Five plants were used for each of 13 treatments in three repetitions (195 plants in total). After inoculation, each treatment was placed into a plastic bag for 48 h to increase the humidity and facilitate penetration of the bacteria into the leaves. Afterwards, the plants were cultivated at 24 °C, 85% humidity, and 16 h of light for 30 days.

**Figure 1:**
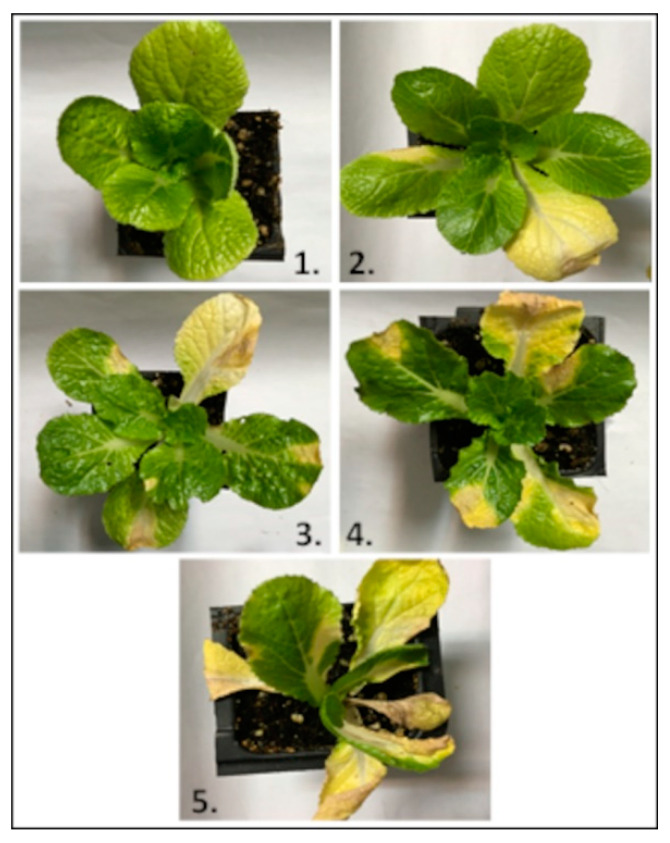
Scale used to evaluate plant reactions to the pathogen. 1—No symptoms. 2—Lesions on 25% of the leaves. 3—Lesions on 25–50% of the leaves. 4—Lesions on 50–75% of the leaves. 5—Lesions on more than 75% of the leaves.

The virulence of each ETS was evaluated based on the reaction of the plant to the infection. The evaluation was based on a scale of 0 to 5 (Figure 1), where the number of infected leaves on each plant was evaluated according to Peňázová et al. [41]. Evaluations were performed twice per week for three weeks (six evaluations in total). Obtained data were analyzed by Statistica 12 software (StatSoft, Prague, Czech Republic) using One-way analysis of variance (ANOVA) with *p* ≤ 0.05. Significant differences with the confidence interval of 95% were detected by applying Tukey’s honestly significant difference (HSD) test.

### 2.3. Assessment of the Stability of Induced Changes in Virulence by Reisolation and Reinoculation

Recultivation of the ETSs from the infected plants was performed 20 days after the first round of inoculation, according to the OEPP/EPPO methodology for *Xanthomonas* spp. [42] with minor modifications. Briefly, symptomatic leaves from plants representing individual ETSs were washed in tap water, rinsed with distilled water, and surface disinfected with 70% ethanol (Lach-Ner, Bratislava, Slovakia). Four small pieces of each leaf were then cut from the bordering necrotic lesion on the leaf and placed into tubes with physiological solution for 1 h to inoculate the solution with the bacteria from the infected tissue. Subsequently, 100 µl of the solution was streaked onto Petri dishes with non-selective Mueller-Hinton agar and incubated at 28 °C for 5 days until the appearance of typical yellow colonies.

The affiliation of overgrown bacterial colonies to a *Xcc* pathogen was verified by PCR reaction, which was performed according to Berg et al. [43] and Eichmeier et al. [44]. Verified *Xcc* colonies were transferred into liquid Mueller-Hinton broth and subsequently used for reinoculation of a new set of *B. rapa* var. *pekinensis* ‘Hilton´ seedlings to evaluate their virulence as described above.

### 2.4. DNA Methylation-Sensitive Profiling of Individual ETSs Using MSAP

DNA from individual ETSs was immediately isolated after treatment using a NucleoSpin^®^ Tissue kit (Macherey-Nagel, Düren, Germany) following the manufacturer’s instructions. Extracted DNA (100 ng) was used as a template for MSAP analysis according to Baránek et al. [45]. Briefly, the isoschizomers *Hpa*II/*Msp*I, which recognize the 5′-CCGG sequence with different sensitivities to the methylation of the “inner and outer” cytosines, and *Eco*RI were used in the restriction reaction. DNA was cut in two reactions, first with a combination of *Eco*RI and *Msp*I, and second with *Eco*RI and *Hpa*II. For selective amplification, three differently labelled primers derived from the *Eco*RI restriction site were used, namely, *Eco*RI-ACA (FAM), *Eco*RI-AGC (NED), and *Eco*RI-ACT (JOE). These labelled primers were used in combination with primers derived from the *Hpa*II/*Msp*I restriction site, namely, *Hpa*II/*Msp*I-TAC, *Hpa*II/*Msp*I-TGC and *Hpa*II/*Msp*I-AGCT. In this manner, 12 different primer combinations were used for PCR. The PCR amplification products were separated by capillary electrophoresis using an ABI PRISM 310 genetic analyzer (Applied Biosystems, Carlsbad, CA, USA). GeneScan 500 ROX (Applied Biosystems, Carlsbad, CA, USA) was used as the size standard, and POP 4 polymer (Applied Biosystems, Carlsbad, CA, USA) was used as the medium for DNA fragment separation. 

The DNA methylation-sensitive profiling of individual ETSs was compared on the basis of the distribution of the MSAP amplicons within individual samples (i.e., the presence or absence of a given DNA fragment) using GeneScan (Applied Biosystems, Carlsbad, CA, USA). To confirm the repeatability of the MSAP results and to assess the internal variability of MSAP profiles occurring within the ETSs, biological replicates were included in the experiment for arbitrary selected variants. 

A binary file reflecting the presence or absence of individual amplicons within the spectra generated by individual ETSs was created, and a similarity degree of the obtained binary data was calculated using the Nei and Li/Dice similarity index [46]. The neighbor-joining method with dissimilarity coefficients was used for data analysis, and a dendrogram was generated with NT-SYS (v. 2.11T, Exeter Software, Setauket, NY, USA).

### 2.5. RNA Extraction, Library Preparation and High-Throughput Dual RNA Sequencing of the Treatments by Using Lomeguatrib and CAY10602 Chemicals

Dual RNA-seq was performed with the intention of revealing the nature of the distinctness of isolates that showed the most different properties on the basis of virulence and MSAP, i.e., the ETSs derived from the Lomeguatrib (LOM) and CAY10602 (CAY) treatments. Seven days after infection, leaf tissue samples (*n* = 4: plant, plant+Xcc WHRI 1279A, plant+Xcc WHRI 1279A treated by CAY, plant+Xcc WHRI 1279A treated by LOM) were ground in liquid nitrogen, and total RNA was extracted using PureLink™ Plant RNA Reagent (Thermo Fisher Scientific, Waltham, MA, USA) with slight modifications [47] to the manufacturer’s instructions. The total RNA yield and quality were measured using a Bioanalyzer 2100 (Agilent Technologies, Palo Alto, CA, USA), using an Agilent RNA 6000 Nano kit and Modulus™ Single Tube Multimode Reader (Turner Biosystems, Sunnyvale, CA, USA), and using a Quant-iT™ RNA Assay kit (Thermo Fisher Scientific, Waltham, MA, USA). Samples with an RNA integrity number (RIN) below 7 were excluded from further analysis. Further quality check, library construction, RNA sequencing, and reads count were outsourced to Vertis Biotechnologie AG (Freising, Germany). Briefly, bacterial and host mRNA were enriched prior to library preparation by ribodepletion using an in-house developed protocol in Vertis Biotechnologie AG. The ribodepleted RNA samples were fragmented using ultrasound (1 pulse of 30 s at 4 °C), after which an oligonucleotide adapter was ligated to the 3′ end of the RNA molecules. First-strand cDNA synthesis was performed using QuantumScript™ Reverse Transcriptase (MCLAB, San Francisco, CA, USA) and the 3′ adapter as a primer. Then, the first-strand cDNA was purified, and the 5′ Illumina TruSeq (Illumina Inc., San Diego, CA, USA) sequencing adapter was ligated to the 3′ end of the antisense cDNA. The resulting cDNA was PCR-amplified to obtain approximately 10–20 ng·µL^−^^1^ of product, using a high-fidelity DNA polymerase (Q5^®^ High-Fidelity DNA Polymerase, NEB, Ipswich, MA, USA), with the number of PCR cycles indicated in Table 2. The TruSeq barcode sequences, which are part of the 5′ and 3′ TruSeq sequencing adapters, are also included in Table 2. The cDNA was purified using an Agencourt AMPure XP kit (Beckman Coulter Genomics, Indianapolis, IN, USA) and assessed by capillary electrophoresis with a Shimadzu MultiNA microchip electrophoresis system. For Illumina NextSeq 500 (Illumina Inc., San Diego, CA, USA) sequencing by synthesis (1 × 75 bp read length), the samples were pooled in equimolar amounts. Then, the cDNA pool was size fractionated at 200–600 bp using a differential clean-up approach with the Agencourt AMPure kit. Then, an aliquot of the size-fractionated pool was analyzed by MultiNA capillary electrophoresis (Shimadzu, Kyoto, Japan).

### 2.6. High-Throughput Sequencing Data Evaluation

The sequence quality was controlled with FastQC-0.10.1 [48]. Trimmomatic-0.36 (usadellab.org) was used to trim and merge the single reads using the following parameters: -phred33; ILLUMINACLIP:adapter:2:30:10; SLIDINGWINDOW:4:15; LEADING:3; TRAILING:3; and MINLEN:30. Remains of both eukaryotic and bacterial rRNA reads were removed by using the SortMeRNA tool version 2.1b (Bonsai Bioinformatics research group, University of Lille, France) [49]. Expression values were calculated with CLC Genomics Workbench 2.0 (CLC Bio, Aarhus, Denmark), and the ‘RNA-seq Analysis’ tool was used with the standard parameters. 

For *B. rapa*, we used the genome with the NCBI ID GCA_000309985.2 (downloaded 5 March 2020) as a reference. Based on the distribution of the raw reads, we filtered out 17,814 contigs (43.6%) that had an average coverage of less than five reads (Figure S2A). According to the abovementioned procedure, we used the r-log normalization and the same downstream analysis. All four r-log normalized samples have normally distributed values (Figure S2B). We selected the top 1% of genes (229 genes) with the highest absolute impact per individual principal component to perform GOEA and PPIEA.

For Xcc, we used the genome with the NCBI ID NC_003902 (downloaded 10 March 2020) as the reference. We excluded 310 contigs possessing reads that mapped to the negative Xcc sample. These contigs were inconsistently covered, often giving a hit with one single read (Figure S1). The omitted reads were identified as the part of tRNAs, rRNAs, pseudogenes and conserved multicopy genes. The raw counts of the three Xcc samples were normalized via regularized log transformation (r-log), implemented in the R package DESeq2, via RPKM and TPM normalization implemented in CLC Genomics Workbench 2.0. Judging from the distributions of normalized values and Pearson’s correlation coefficients of replicate-replicate comparisons (Figure S1), we continued with the rlog values. The normalized values were used for principal component analysis (function prcomp in R) to identify genes with the highest impact on sample-specific differences. We took 5% of the genes (116 genes) with the highest absolute value per individual principal component and used them as input for the gene ontology enrichment analysis (GOEA) and protein-protein interaction enrichment analysis (PPIEA), which was performed using a ShinyGO graphical gene-set enrichment tool for plants [50] and the STRING DB.

Unexpectedly high differences in the similarity coefficients obtained by the MSAP analysis of tretaed Xcc (see results) were the impetus for assessing whether, in addition to differences in DNA methylation, point mutations also contribute to the observed differences. To call SNPs, we used SAMtools (version 1.7) [51], where the function mpileup with parameters-C 50-d 100-Q 15 was used. Inputs for the SNP calling were bam files produced during the RNA-seq reads mapping. First, we corrected reference genomes of Xcc (NC_003902) for point mutations and short indels present in our ancestral strain (the negative control) with a custom-designed script (written in bash), to minimize the amount of falsely called SNPs. Subsequently, we compared the positive sample and strains after Lomeguatrib (LOM) and CAY10602 (CAY) treatments against the adjusted reference genome. We applied a loose SNP filtering. An SNP was kept if it is covered by more than one read pair (the total depth must be bigger than one) and the genotype must be 0/0 (homozygous). Motifs applied for calling (custom-designed script was utilized) were used on the basis of their presence in other Xanthomonas species [24,52,53] or to have covered motif targeted by endonuclese used in MSAP analysis.

## 3. Results

### 3.1. Virulence of Individual ETSs and Stability of Induced Changes as Revealed by Reisolation and Reinoculation of New Sets of Plants

**Figure 2:**
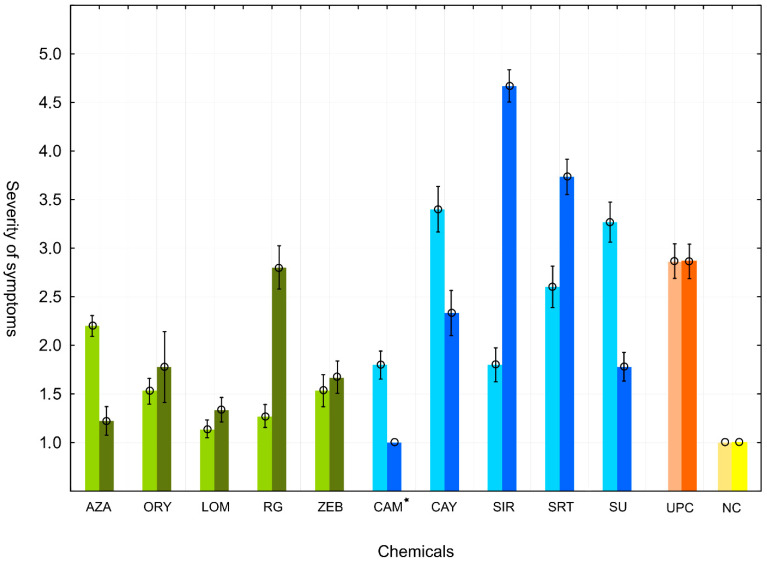
Impact of individual epigenetic modulating chemical treatments on the virulence of ETSs 15 days after the inoculation of *B. rapa* plants. Bright columns of the respective color represent virulence of the strains used immediately after treatment. Dark columns of the respective color represent virulence of the strains reisolated from the plants 20 days after the first round of inoculation. Means of three independent repetitions are presented, and the standard deviation is indicated as error bars. UPC = untreated positive control (inoculation by untreated *Xcc* strain); NC = negative control (no *Xcc* was applied on plants); * = unconfirmed presence of *Xcc* in the CAM-derived ETS after the second round of inoculation.

Treatment with individual EMCs usually had a significant impact on virulence, compared to the untreated *Xcc* strain control variants (see symptoms severity in Figure S3 and results of Tukey’s HSD test in Table S1). The comparison of virulence scores is summarized in Figure 2 (bright columns), where the values obtained when symptoms began showing in ETS treated plants (15 days after inoculation). To confirm the viability of the treated strains and evaluate the longevity and repeatability of the initial effects on virulence, the individual strains were reisolated from the initially inoculated plants, cultivated in standard liquid medium and then used as inocula in the second round of inoculations. The virulence observed for the individual ETSs in the second round of inoculations is summarized also in Figure 2 (dark columns).

In the first round of inoculations, most of the EMCs (AZA, ORY, ZEB, RG, SIR, CAM, and LOM) caused decreased virulence compared to that observed in the untreated control. The most significant decrease in virulence was observed for the ETSs derived from the LOM and RG treatments. Compared to the control, only the ETS derived from the CAY treatment had a significant increase in virulence.

In the second round of inoculations, most of the reisolated ETSs derived from the EMC treatments (AZA, ORY, ZEB, SU, CAM, CAY, and LOM treatments) retained their decreased virulence, compared to the positive control. In contrast, the ETSs derived from the SIR and SRT treatments showed significantly increased virulence compared to the control after reisolation.

Regarding the repeatability and stability of the effect on virulence, a comparison between the first and second rounds of inoculation (Figure 2) revealed some promising candidates with seemingly durable modulated properties. It is important to emphasize that the ETS derived from the LOM treatment showed extremely low virulence, similar to negative control, for both rounds of inoculations. A similar phenotype was also observed for the ETSs derived from the ORY and ZEB treatments. In contrast, no ETS showed increased virulence in both rounds of inoculation. The CAY treatment-derived ETS from the first round and the SIR and SRT treatment-derived ETSs from the second round were identified as having significantly higher virulence than the control.

### 3.2. Effect of the EMCs on the DNA Methylation-Sensitive Profiling of the Treated Strains

**Figure 3:**
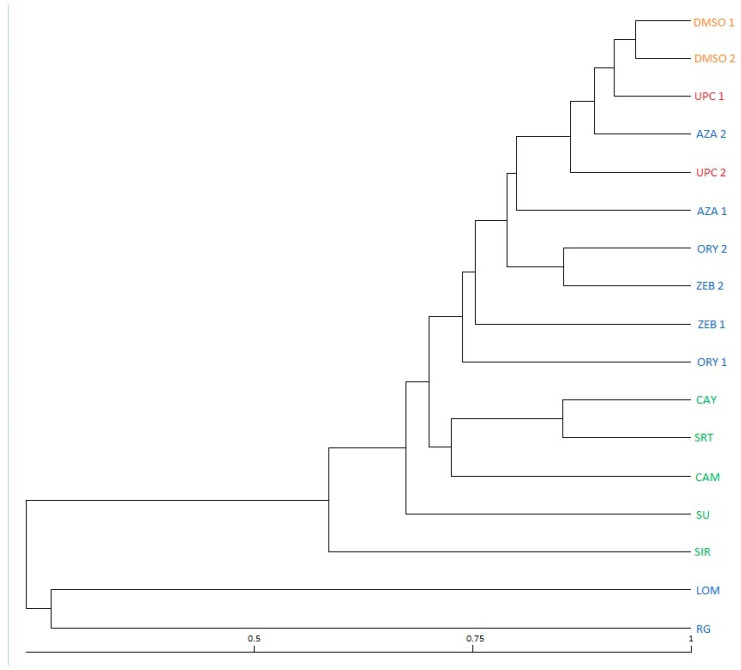
Dendrogram depicting similarities between DNA methylation-sensitive profiles of individual strains treated by respective epigenetic modulating chemicals. Numbered samples represent biological replicates of individual variants to control their internal variability.

A dendrogram constructed based on the MSAP analysis (Figure 3) split samples into a few distinct clusters. The LOM and RG treatment-derived ETSs formed the most separated clade and clearly differed from the rest of the samples. The SIR treatment-derived ETS was also distinguished from other ETSs, but not as significantly as observed for the LOM and RG treatment-derived ETSs. A group of internal biological controls included in the analysis confirmed that the profiling of the samples derived from same ETSs showed slight differences but retained sufficient stability for credible comparisons of the effects of the *Xcc* WHRI 1279A treatments performed in this study. Because the resulting coefficients of similarity were surprisingly low between some treatments, the data from dualRNA sequencing were further controlled for the frequency of point mutations as potential contributors to the observed differences in MSAP profiles (the results of SNP calling are shown below).

### 3.3. Identification of the Contigs with the Highest Impact on the Variability within Xcc Transcriptomes Evaluated within Dual RNA-Seq

The sequencing data are available in the NCBI database through BioProject ID PRJNA615172 and BioSample accessions SAMN14447500, SAMN14447501, SAMN14447502, and SAMN14447503. Additional data processing details are provided in Figure S1.

**Figure 4:**
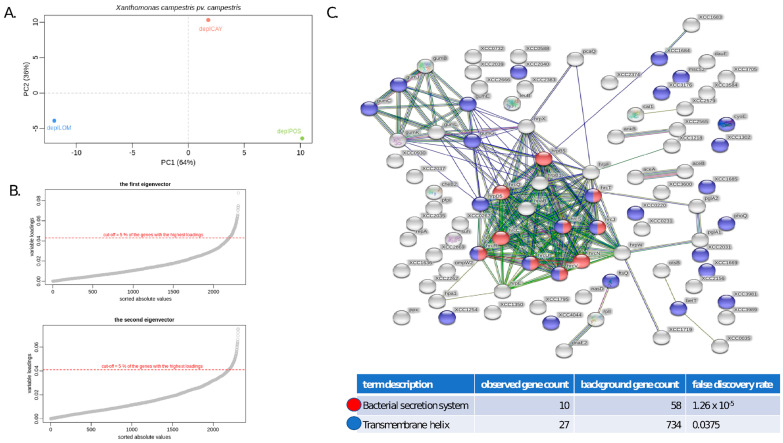
Characterization of differentially expressed contigs within the analyzed *Xcc* WHRI 1279A variants. (**A**) PCA of normalized altered transcriptomes of positive (deplPOS), deplCAY- and deplLOM-treated *Xcc* variants during their infection of *B*. *rapa* plants (**B**) Selection process for contigs from the first (deplPOS + deplCAY vs. deplLOM *Xcc* strains) and the second (deplCAY vs. deplLOM + deplPOS) eigenvector at the level of 5%. (**C**) Enriched KEGG pathways identified as having the highest impact on the differences between the deplLOM and (deplCAY + deplPOS) transcriptomes.

In the PCA, the first principal component captured 64% of the variability of the normalized filtered transcriptomes, while the second principal component captured 36% of the variability. The sample originating from plants infected with the LOM treatment-derived ETS (deplLOM) was strictly separated from the CAY treatment-derived (deplCAY) and the positive control (deplPOS) samples, based on the first principal component (Figure 4A). The second principal component (Figure 4A) shows the samples deplPOS and deplLOM being closer together and shifts the sample deplCAY away (along the Y-axis). Of the 116 genes representing the top 5% of genes with the highest absolute values in the first eigenvector, 28 genes represent tRNAs, and the remaining 88 are protein coding genes. These 88 genes display enrichment in protein-protein interactions (PPIs; *p*-value < 1 × 10^−16^, 193 edges), with the most significant KEGG pathway recognized as “bacterial secretion system” (FDR= 1.26 × 10^−5^; Figure 4C). Of the 116 genes with the highest absolute values in the second eigenvector, 82 are protein coding, and the remaining ones represent tRNAs sequences. These 82 genes contain 32 edges (32 predicted protein-protein interactions, *p*-value < 0.168), but no significantly enriched gene ontology term or KEGG pathway was identified.

### 3.4. Identification of the Contigs with the Highest Impact on the Variability of B. rapa Transcriptomes Evaluated within Dual RNA-Seq

**Figure 5:**
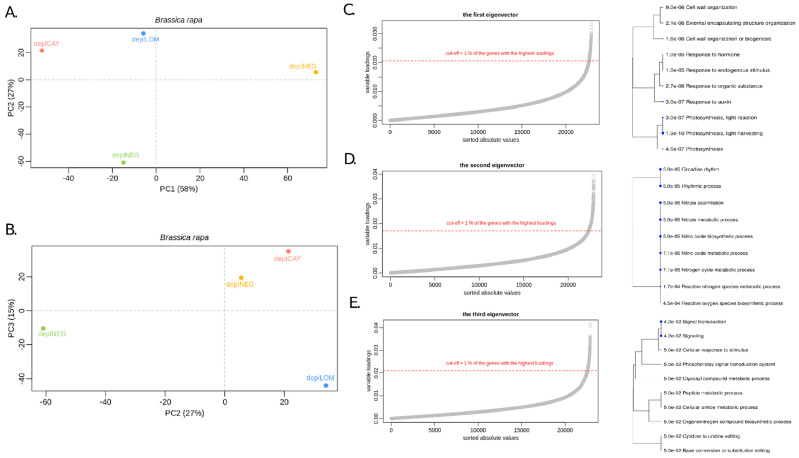
Characterization of differentially expressed contigs in the transcriptomes of the analyzed variants of *B. rapa* after infection by individual *Xcc* WHRI 1279A variants. (**A**) PCA of the normalized altered transcriptomes of *B*. *rapa* plants infected by individual *Xcc* variants (1st and 2nd eigenvectors). (**B**) PCA of normalized altered transcriptomes of *B*. *rapa* plants infected by individual *Xcc* variants (2nd and 3rd eigenvectors). (**C**) Selection process of the contigs from the first eigenvector (distinguishing the negative sample deplNEG from the rest) at the level of 1% and respective enriched gene ontology analysis. (**D**) Selection process of the contigs from the second eigenvector (distinguishing the positive sample deplPOS from the rest) at the level of 1% and respective enriched gene ontology analysis. (**E**) Selection process of the contigs from the third eigenvector (distinguishing the deplCAY sample from the rest) at the level of 1% and respective enriched gene ontology analysis.

PCA reduced four analyzed samples into three significant components (Figure 5). The first principal component captures 58%, the second nearly 27%, and the third nearly 15% of the variability. Samples from two clusters (deplPOS, deplCAY, deplLOM versus deplNEG) along the x-axis (Figure 5A) i.e., first principal component. Towards the second principal component (Figure 5A,B) are the samples divided into other groups: deplPOS versus deplCAY, deplLOM (deplNEG as negative control is close to zero). The third principal component captures the variability between sample deplCAY and the rest of samples, where contribution of deplLOM is substantial (contributions of the deplPOS and deplNEG are small). Here, for each of the three significant components, 1% of the contigs represented by 229 of them (219 protein coding genes in the case of the first eigenvector, 216 protein coding genes in the case of the second eigenvector, and 132 protein coding genes in the case of the third eigenvector) with the highest absolute values of variability were used to perform gene ontology enrichment analysis (GOEA) (Figure 5C–E) for each of the three eigenvectors.

### 3.5. Control ofthe Dual RNA-Seq Data in Terms of Point Mutations in Xcc Genomes after Treatments by Using Lomeguatrib and CAY10602 Chemicals

**Figure 6:**
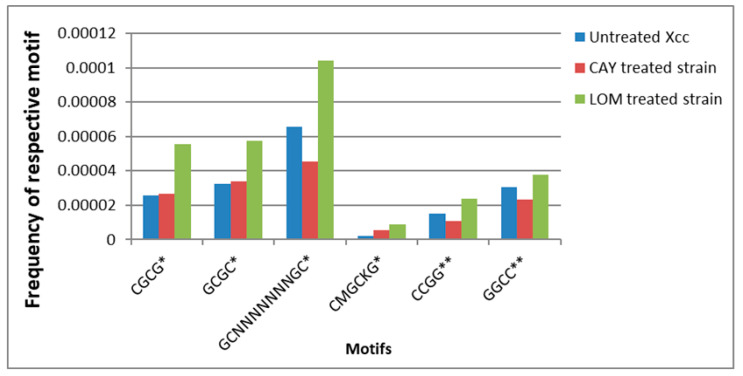
Results of SNPs calling within potentially methylatable motifs by using dual RNA-seq data. * = motif usually associated with m4C methylation; ** = motif usually associated with m5C methylation; respective complementary sequences are also included in depicted frequencies.

The overall frequency of point mutations registered by comparison of dualRNA sequencing data with reference to genomes are given in the Table S2. The results of the comparison of SNPs’ frequency within specific motifs previously described as potentially methylatable in the Xanthomonas genome are presented in Figure 6. It is evident that Lomeguatrib treatment induced the SNPs in the screened motifs generally more often. Specifically, the biggest difference in SNPs’ occurrence between variants was registered for the motif CMGCKG, where SNPs in LOM-derived treatment were 4.32 times higher than in the untreated control. Regarding CCGG motif recognized by methylation sensitive endonucleases used in MSAP protocol were registered SNPs in LOM-derived treatment 1.58 times more frequently than in the untreated control. In general, higher mutation values registered for LOM treated strain should be taken into account when discussing the observed change in virulence and differences in MSAP profiles. In both cases, the observed properties were probably influenced by the combined effect of altered methylation and subsequent transformation of some sites with altered methylation to SNPs, due to the cumulative effect of their higher tendency for mismatch pairing and an affected mismatch repair system. Unfortunately, these phenomena are consecutive and difficult to distinguish from each other (for details, see discussion).

## 4. Discussion

### 4.1. Overall Evaluation of the Effect of EMCs on the Properties of Treated Xcc Strain

To date, only two studies have investigated chemical epigenetic modifications in bacteria. Kumar et al. [54] studied the epigenetic activation of antibacterial properties of the endophytic Streptomyces coelicolor Waksman & Henrici after azacytidine treatment. Yadav et al. [55] reported the 5-azacytidine inhibited in vitro biofilm formation and decreased the expression of genes involved in the methionine recycling pathway. The results of the present study confirm changed properties after the treatment by epigenetic modulating chemicals by monitoring virulence, DNA methylation-sensitive profiling, and transcriptomic analyses. Additional analysis demonstrated that treatment with DNA demethylating agents also affects the frequency of point mutations. In fact, a higher proportion of SNPs in a strain treated with Lomeguatrib was registered if methylatable motifs are considered. This is not necessarily surprising in light of the fact that methylation significantly increases the frequency of mismatch pairing during replication [56] and DNA methylation at a given site is an important marker for a bacterial DNA mismatch repair system [10,16]. For example, increased levels of methylated nucleotide mutability have been observed for 5-methyl-cytosines [57] but also for 6-methyl adenine [58]. More specifically, comprehensive comparison of thousands of bacterial genomes showed that DNA methylation increases the mutational rate 12 to 58 times in screened species [57]. Artificial changes in DNA methylation, as performed by treatment used in this study, can therefore also cause a significantly higher mutation rate. However, at the same time, it must be acknowledged that the question of the nature and mechanism of point mutations induced after EMS treatment remains underexplored. More focused experiments to solve these gaps in knowledge are therefore needed and necessary for smarter use of chemically induced changes in DNA methylation to modulate properties of bacteria.

However, it is clear from the above that the treatment of bacterial cultures by EMC probably induces a process of DNA demethylation a first, to which, in a certain incidence, the process of point mutation of a given site is directly linked. This means that changed bacterial properties being evaluated in this study should be also considered as a consequence of the interconnected effect of DNA demethylation and subsequent point mutations.

### 4.2. Virulence of Individual ETSs on B. rapa Plants

Most of the observed significant differences in virulence compared to the untreated control were decreases in virulence. Interestingly, all of the five assayed EMCs with hypothetical DNA demethylation effects decreased the virulence of the treated strain in both rounds of inoculations (Figure 2). The observation of decreased virulence in the case of lowered DNA methylation was also directly or indirectly observed in other studies [59,60,61]. However, the principles of these experiments were quite different, because strains with a mutant *DNA adenine methyltransferase* (*Dam*) gene were used to induce DNA methylation decreases. Very recently, it was demonstrated that global change in DNA methylation affects so-called phasevarions switching (phase-variable regulons), which alters bacterial virulence of human pathogens [62]. As indicated above, the effect of lowered virulence observed in this study could also be supported by the event of mutations appearing post DNA demethylation, which can generally also affect virulence if it occurred in the causal gene [63,64].

The effect of EMCs that affect sirtuins were very difficult to predict because the biological roles played by sirtuins in bacteria are still not well described. However, it is already known that de-acetylation caused by the best characterized bacterial sirtuin CobB activates the metabolic enzyme acetyl coenzyme-A synthetase (ACS) in *Salmonella enterica* Le Minor & Popoff [65] and affects the activity of *N*-hydroxyarylamine *O*-acetyltransferase (NhoA) in *Escherichia coli* Castellani & Chalmers [66]. CobB has also been linked to signal transduction and transcriptional regulation in *E. coli*, where it regulates chemotaxis by deacetylating the response regulator CheY [67], modulates the transcription of *cpxP* from its stress-responsive promoter by deacetylating the α-subunit of RNA polymerase [68], and promotes transcription of the RcsC regulon by de-acetylating the transcription factor RcsB [69,70].

Regarding the sirtuins and the effects of their treatment on respective ETS virulence, it is important to note that the effect was varied, where the modified virulence caused by sirtuins in the first round of inoculation was not typically confirmed in the second round of inoculation (after the reisolation of treated strains from plants). However, all of the cases when the virulence was increased, compared to the control involved ETSs, derived from the sirtuin-modifying treatment. Thus, it is possible to summarize that treatment by sirtuin-modifying compounds showed some interesting properties that should be investigated in more focused experiments in future studies.

### 4.3. Results of DNA Methylation-Sensitive Profiling of the Strains Treatedby Individual EMCs

As previously mentioned, the results obtained by using the MSAP method (see Figure 3) were the impetus for the additional evaluation of the obtained sequencing data, because the low values of similarity coefficients derived from MSAP spectra suggested that another factor, such as SNPs, could play role here. Based on the confirmation of a higher incidence of SNPs in the variant where the DNA demethylating chemical was used, it is necessary to admit that the observed polymorphism reflects both different methylation in the CCGG recognition sequence and a possible mutation of this sequence by transition of methylated cytosine to thymine. Here, it is necessary to remember that due to the significantly higher mutability of methylated cytosines [57], however, cases of polymorphism caused by a mutation in the recognition sequence are still primarily caused by a change in cytosine methylation induced by a given treatment.

The most different group of ETSs within the generated dendrogram (Figure 3), in terms of their DNA methylation-sensitive profiles, were those treated by Lomeguatrib and RG108 chemicals. Both EMCs have previously demonstrated DNA demethylating effects, and both caused the most significant decrease in virulence in the first round of inoculations (Figure 2). Another group with different DNA methylation-sensitive profiles were ETSs treated by Sirtinol, SRT 1720, Cambinol, CAY10602, and Suramine. All EMCs from this group have demonstrated effects on sirtuins, which deacetylate cell proteins. How the acetylation of a cell protein is linked with DNA methylation in bacteria remains unknown. In eukaryotic organisms, we have examples where a mutual dependence exists between histone acetylation and DNA methylation in the process of gene silencing [71,72]. However, a recent study reported that bacterial protein acetylation had a prominent role in central and secondary metabolism, virulence, transcription, and translation [22], where these observed properties could be controlled, at least in some cases, also by DNA methylation.

Within the largest cluster of the generated MSAP dendrogram were variants representing the positive control, the DMSO-treated variant and, surprisingly, the ETS treated with azacytidine. Azacytidine is typically used as a “gold standard”, with the vast majority of studies focused on inducing DNA demethylation using chemicals. A possible explanation of this weak effect is that azacytidine solutions are not very stable. A better alternative is to use Zebularine, which has a demonstrated higher stability, or analogues of azacytidine with higher stability/effect [73].

Biological repeats for various ETSs were also added to the MSAP analysis to verify the results, where the level of variability of all biological replicates was satisfactorily low, and all repeats were concentrated in the same cluster.

### 4.4. Comparison of Xcc and B. rapa Transcriptomes by Dual RNA-Seq

Different characteristics of two significantly altered ETSs were also confirmed through a dual RNA-seq analysis. Comparisons of *Xcc* WHRI 1279A transcriptomes confirmed the distinctness of the LOM treatment-derived ETS from the CAY treatment-derived ETS and the untreated strain, which corresponds to their virulence assay results and those of the MSAP analysis. The dual RNA-seq results also shed light on the molecular background of the observed differences with respect to virulence. Enriched KEGG analysis results identified the genes representing “bacterial secretion system” as the most different between deplPOS + deplCAY vs. deplLOM samples. Indeed, the vast majority of genes in the most different cluster belongs to the group *hrp* (hypersensitive reaction and pathogenicity) and *hrc* (hypersensitive response conserved) genes, representing genes involved in the type III secretion system (T3SS), which is crucial for the pathogenesis of *Xanthomonas* pathogens [74,75]. Therefore, a direct impact of the observed differences in the expression of these genes from this group on virulence for the respective ETSs is highly evident.

KEGG pathway analysis (Figure 4C, the upper left corner) also highlighted the importance of the gum operon. Seven of twelve gum genes passed the criterion of having the highest impact on the differences between the LOM and (CAY + positive) samples, factually all seven respective genes had lower expression than in CAY or positive samples. The genes of the gum operon family, a 14-kb gene cluster comprising 12 CDSs, named gumBCDEFGHIJKLM [76,77], perform the biosynthesis of xanthan, which aids in the attachment of pathogenic *Xanthomonas* species to plant surfaces via biofilm formation. Therefore, xanthan is essential for the successful colonization and growth of *Xcc in planta*, and genes from this group are often described as having an essential role in pathogenicity [78,79]. In summary, our results comparing the transcriptomes of variants with different virulence characteristics confirmed that genes involved in the type III secretion system and its regulation, together with genes from gum pathways, are good targets for studying and modifying the pathogenicity of *Xcc*.

A comparison of the *B. rapa* transcriptomes on PCA1 showed that the most contrastingly expressed clusters of genes are responsible for: (i) responses to different stimuli; (ii) genes involved in the cell wall organization; and (iii) genes involved in photosynthesis (see Figure 5C). Because the most diverse variant on PCA1 represents the negative control and because general principles of how plants react to bacterial pathogens are well known, the identification of the most differentially expressed clusters in infected plants is not surprising. Enriched gene ontology terms based on the 2nd principal component analysis (divide deplPOS versus deplCAY and deplLOM) showed a group of genes regulating nitrogen metabolism as being the most notable (see Figure 5D). Nitrogen is traditionally recognized as essential for life and is a limiting factor of plant growth. However, many agronomic data indicate that nitrogen supply has a positive impact on the incidence of crop diseases. Although it has been known for a long time that nitrogen availability affects disease, the underlying mechanisms were only recently hypothesized [80]. In general, it is evident that nitrogen influences both constitutive and induced defense systems, and is a key element in signal molecules such as nitric oxide and polyamines [81]. In addition to nitrogen-associated genes, some other groups of genes that are well known to be important in plant-pathogen interactions (reactive oxygen, circadian rhythm) were identified in the corresponding 2nd PCA ontology. The third PCA captured mainly the variability between samples deplCAY and deplLOM (contributions of the deplPOS and deplNEG are small), which represented the most diverse virulence within the first round of inoculation. Genes differentially expressed within the third PCA were included in biological rhythms regulation and the signal transduction process, which are generally crucial in the first phases of plant reaction to stress [82,83].

## 5. Conclusions

The present study represents the first genuine investigation of the effects of different epigenetic modulating chemicals on phytopathogenic bacteria. Treatment with DNA demethylating chemicals produced unambiguous effects, where all treated strains showed decreased virulence. The use of compounds to alter sirtuin activity that in turn modulated the properties of bacteria were evaluated in this study for the first time. Despite only slight effects, when compared to the DNA demethylating EMCs, the results should not be overlooked. 

An aspect of problematic linkage of the phenomenon of altered DNA methylation and subsequent transition of part of such altered bases on another basis (induction of SNPs) was also considered in this work. This fact should be taken into account in similarly oriented studies and the methodology used should be adapted to capture both DNA methylation and SNPs by approaches directly evaluating these two phenomena. In this regard, NGS methods, such as Oxford Nanopore and or PacBio with platforms capable of recognizing methylated and unmethylated bases, seem to be very usable. Generally, the application of epigenetic modulating chemicals may allow for an increased diversity of properties and variants in a given bacterial organism and can contribute to the further development of studies in the field of bacterial epigenetics, ecology and adaptation.

## Figures and Tables

**Table 1:** List of chemicals used for the treatment of *Xcc* reference strain WHRI 1279A.

Chemical/Abbrv.	Demonstrated/Hypothetical Epigenetic Effect	References
Azacytidine/AZA *	Nucleoside analogue of cytidine specifically inhibiting DNA methylation by trapping DNMTs	[25,26]
γ-Oryzanol/ORY *	DNMT inhibitor activity, the mechanism of action needs to be further deciphered	[27,28]
Lomeguatrib/LOM *	Modified guanine base inhibiting the activity of DNA repair protein O(6)-alkylguanine-DNA alkyltransferase	[29,30]
RG108/RG *	Non-nucleoside inhibitor of DNMTs lacking the human cells toxicity	[31,32]
Zebularine/ZEB *	DNA demethylation activity by stabilizing the binding of DNMTs to DNA	[33,34]
Cambinol/CAM **	SIRT1 and SIRT2 inhibitor	[35,36]
CAY10602/CAY **	SIRT1 activator	[37]
Sirtinol/SIR **	SIRT1 and SIRT2 inhibitor	[38]
SRT1720 Hydrochloride/SRT **	Strong SIRT1 and weak SIRT2 and SIRT3 inhibitor	[39]
Suramine/SU **	SIRT1 and SIRT5 inhibitor; potential inhibitor of bacterial RecA protein	[40]

*—confirmed or hypothesized effect on DNA methylation. **—described effect on sirtuin activity.

**Table 2:** Description of cDNA samples.

No.	Sample	PCR Cycles	i5 Barcode	i7 Barcode
1	plant	16	AGGCTTAG	GAGATTCC
2	plant+Xcc (1279A)	16	ATTAGACG	GAGATTCC
3	plant+Xcc (1279A) treated by CAY	16	CGGAGAGA	GAGATTCC
4	plant+Xcc (1279A) treated by LOM	16	CTAGTCGA	GAGATTCC

## Data Availability

All the data are included in this paper and in Supplementary data.

## Supplementary Materials

The following are available online at https://www.mdpi.com/article/10.3390/genes12060804/s1, Figure S1: Intermediates from the evaluation of sequencing data related to *X. campestris* pv. *campestris* (Xcc) genome, Figure S2: Intermediates from the evaluation of sequencing data related to B. rapa genome, Figure S3: Divergent virulence of some epigenetically treated strains of *X. campestris* pv. *campestris* registered 15 days after inoculation, Table S1: Significance of differences in virulence between the individual treatments as revealed by Tukey’s (HSD) test, Table S2: Incidence of point mutations in RNAseq data obtained for strains treated by selected EMC based on the comparison with reference genomome of *X. campestris*
