# Supplementary material for: Epigenetic Modulating Chemicals Significantly Affect the Virulence and Genetic Characteristics of the Bacterial Plant Pathogen Xanthomonas campestris pv. campestris
Source: Genes (Basel). 2021 May 25;12(6):804. doi: 10.3390/genes12060804 (PMC8226645; doi:10.3390/genes12060804)
Supplement: Supplementary file 1 [file genes-12-00804-s001.zip › Figure S1_S2_S3.pdf]

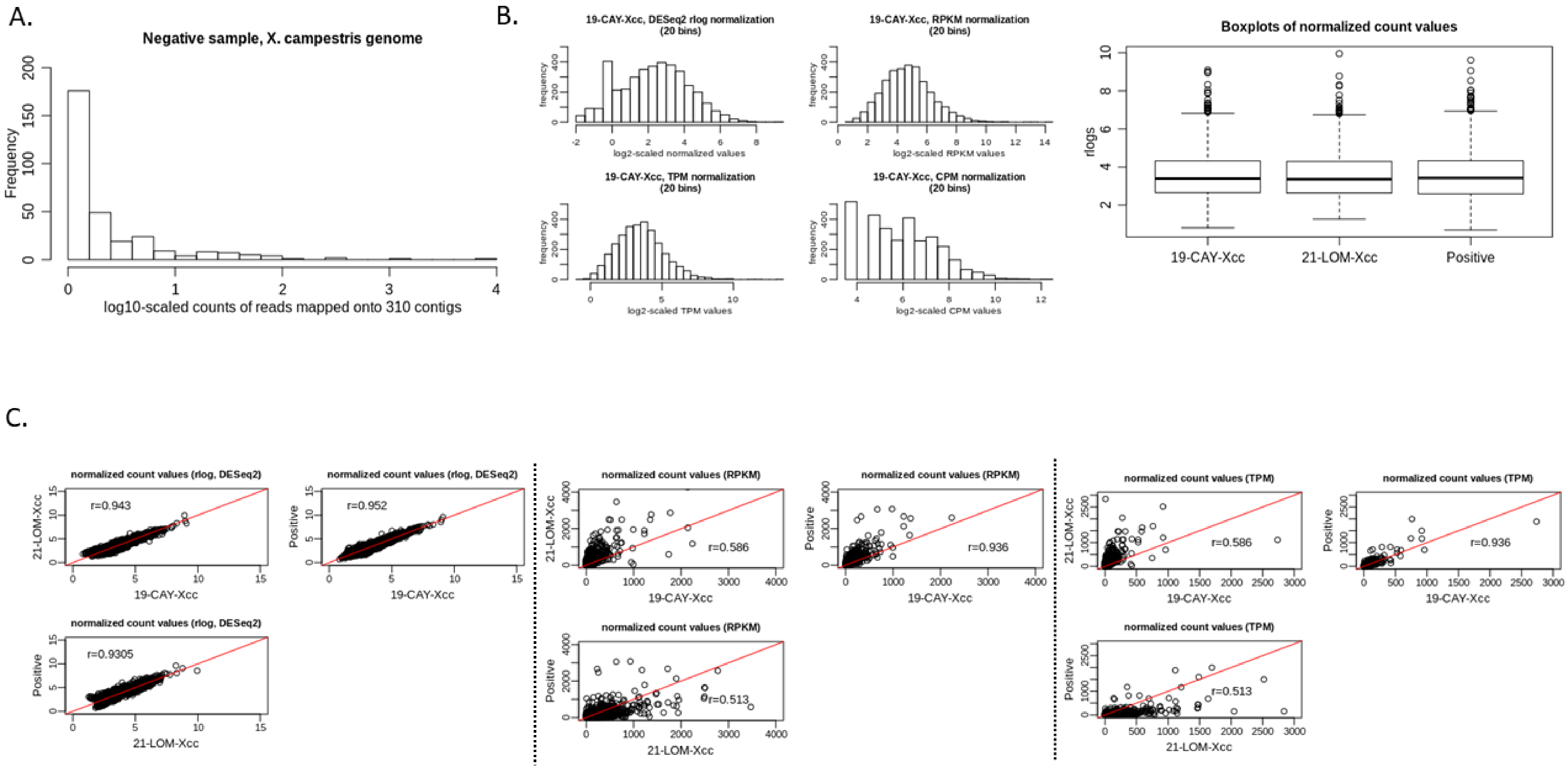

Figure S1. Intermediates from the evaluation of sequencing data related to *X. campestris* pv. *campestris* (*Xcc*) genome. **(A)** Occurrence of 310 contigs that possess mapped reads in the negative *Xcc* sample. **(B)** Comparison of four normalisation methods and rlog normalized counts of reads mapped within individual *Xcc* variants **C** normalized counts of mapped reads for individual *Xcc* variants as generated by individual normalization methods.

A.

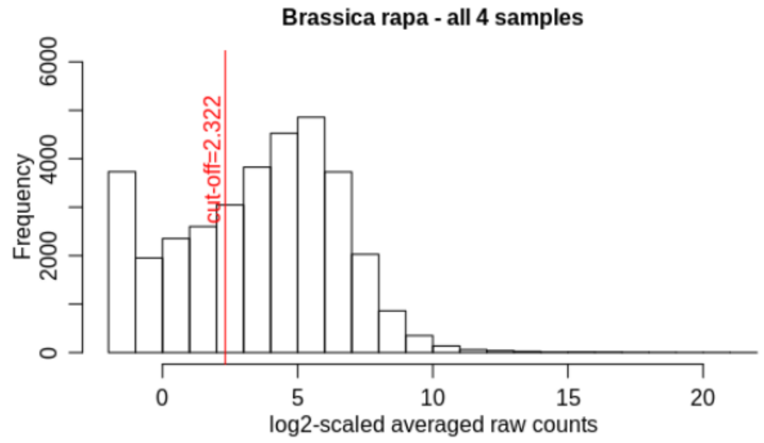

B.

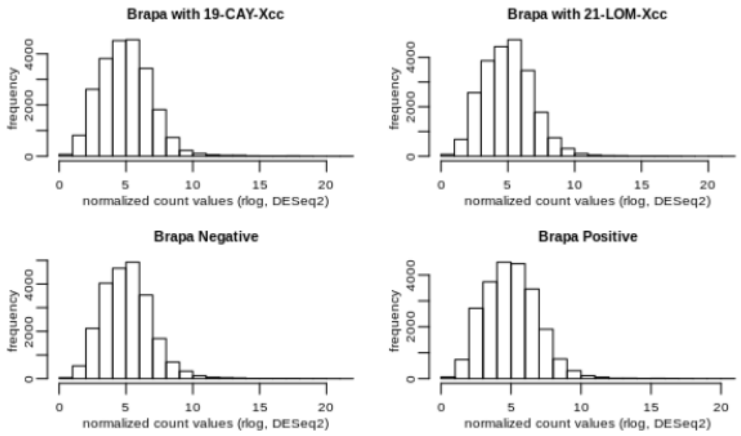

Figure S2. Intermediates from the evaluation of sequencing data related to *B. rapa* genome. **(A)** log2-scaled mean values of mapped reads per contig. (**B)** distributions of rlog normalised values computed for individual variants.

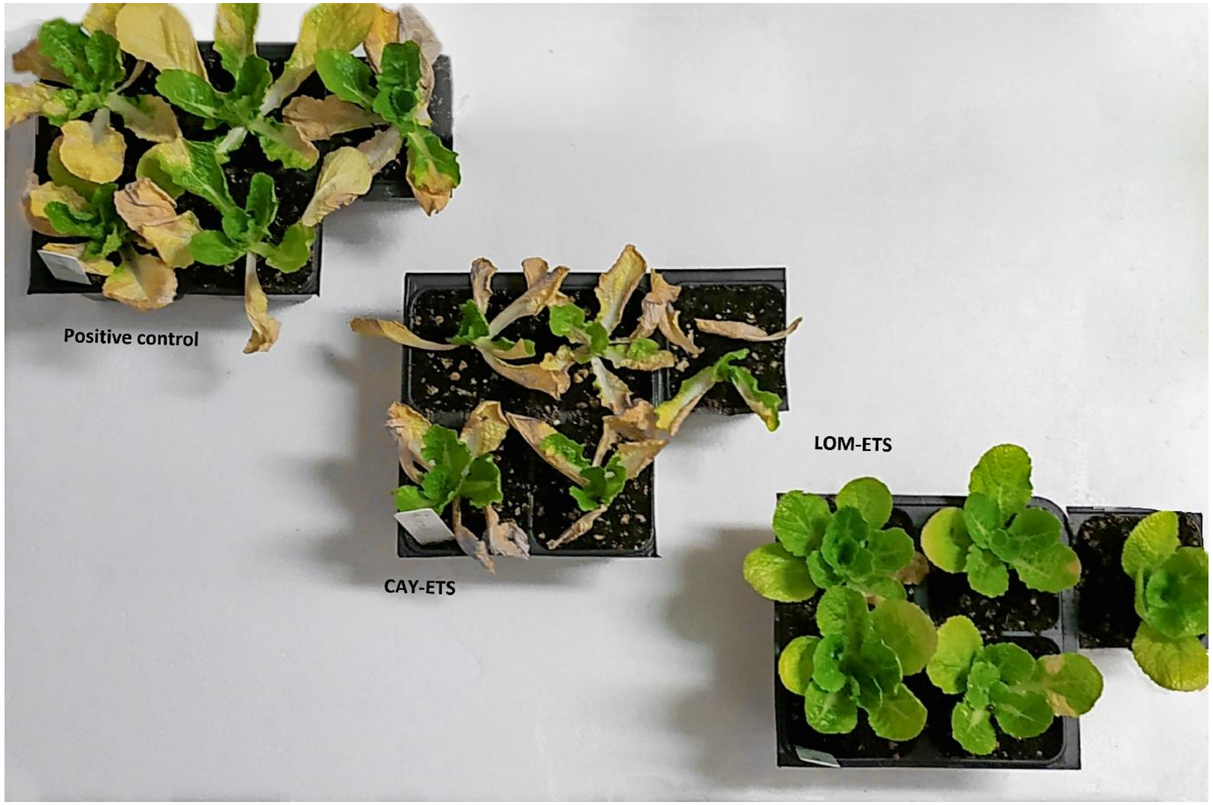

Figure S3. Divergent virulence of some epigenetically treated strains of *X. campestris* pv. *campestris* registered 15 days after inoculation. Positive control represent plants inoculated by untreated Xcc strain, remaining quintuplets represents plants inoculated by Xcc strains treated by CAY and LOM chemicals.
